# A retrospective comparative diagnostic evaluation of three commercial qPCR kits for the diagnosis of mpox virus on an enriched sample set

**DOI:** 10.3389/fcimb.2026.1831182

**Published:** 2026-09-11

**Authors:** Darwin Paredes-Núñez, Silvia Salgado, Martha Sánchez, Katherine Natalia Chávez-Toledo, Johanna Parrales, Andrés Tinizaray, José Julián Zúñiga-Velarde, Solón Alberto Orlando, Miguel Angel Garcia-Bereguiain

**Affiliations:** 1 One Health Research Group, Universidad de Las Américas, Quito, Ecuador; 2 Instituto Nacional de Salud Pública e Investigación, Guayaquil, Ecuador; 3 Universidad Católica Santiago de Guayaquil, Guayaquil, Ecuador; 4 Universidad Ecotec, Samborondón, Ecuador

**Keywords:** clinical performance, commercial kits, mpox, mpox virus, qPCR, WHO

## Abstract

Mpox virus (MPXV) is an enveloped double-stranded DNA virus belonging to the genus *Orthopoxvirus* within the family *Poxviridae*. It causes a zoonotic disease transmitted to humans through direct contact with infected animals or humans, as well as through exposure to contaminated materials. The aim of this study was to conduct a retrospective, comparative diagnostic evaluation of three commercial real-time PCR (qPCR) kits for the diagnosis of MPXV on an enriched, artificially balanced sample set that included 50% MPXV-positive samples. World Health Organization (WHO) reference molecular protocol was used as the reference comparator. A total of 300 clinical specimens, 150 of them MPXV-positive (265 skin lesion/vesicle swabs and 35 serum samples), were used in this analysis. The Bioperfectus kit correctly identified 294/300 samples, showing a sensitivity of 0.96 (95% CI: 0.93–0.99), a specificity of 1.00 (0.98–1.00), and a Cohen’s Kappa coefficient of 0.96 (95% CI: 0.92–0.99), with an overall agreement of 0.98 (0.96–0.99). The VIASURE kit correctly detected 293/300 specimens, yielding a sensitivity of 0.95 (0.92–0.99), specificity of 1.00 (0.98–1.00), Cohen’s Kappa coefficient of 0.95 (95% CI: 0.92–0.99 and an overall agreement of 0.98 (0.95–0.99). The Singuway kit showed the highest concordance with the reference method, correctly classifying 295/300 samples and presenting a sensitivity of 0.97 (0.94–1.00), specificity of 1.00 (0.98–1.00), Cohen’s Kappa coefficient of 0.97 (95% CI: 0.94–1.00), with an overall agreement of 0.98 (0.97–1.00). This study demonstrates a good sensitivity and specificity, and strong overall agreement among the three commercial kits compared to the WHO protocol. Nevertheless, further analysis using unbiased clinical sample sets that reflect the true prevalence of MPXV-positive samples is needed for a real-world clinical performance evaluation.

## Introduction

The mpox virus (MPXV) is an enveloped double-stranded DNA virus. It belongs to the *genus Orthopoxvirus* in the family *Poxviridae*, which causes a zoonotic disease called mpox. The *Orthopoxvirus* genus also includes Vaccinia virus, Cowpox virus, Rabbitpox virus, Variola virus, and several other animal-related poxviruses (Brown and Leggat, 2016; Adalja and Inglesby, 2022). Two phylogenetically distinct clades of MPXV have been identified through genomic sequencing: the Central African Clade I (Congo basin) clade and the West African Clade II. Typically, Clade I MPXV is associated with more severe disease, higher mortality, and more frequent human-to-human transmission (Brown and Leggat, 2016; Chaix et al., 2022; Mohamed Abdoul-Latif et al., 2024).

MPXV is transmitted to humans through contact with an infected animal or human, or with material contaminated with the virus. The virus enters the body through broken skin, the respiratory tract, or the mucous membranes. Virus transmission through direct or indirect contact with live or dead animals is assumed to be the main factor for human mpox infections. This may occur through bite or scratch wounds, bushmeat preparation, direct contact with body fluids, or lesions from an infected animal or contaminated material (indirect contact). Eating inadequately cooked meat from an infected animal is another possible risk factor. As with smallpox, human-to-human transmission of MPXV occurs primarily through large respiratory droplets during direct, prolonged face-to-face contact. In addition, MPXV can be transmitted by direct contact with body fluids of an infected person or with contaminated objects (fomites), such as bedding or clothing. The incubation period of this virus is usually 6–13 days, but can range from 5 to 21 days (Li et al., 2006; Brown and Leggat, 2016; Alakunle et al., 2020; Adalja and Inglesby, 2022).

Human mpox often begins with a combination of the following symptoms: fever, headache, chills, exhaustion, asthenia, swollen lymph nodes, backache, and muscle aches. Commonly, within 1 to 3 days after the onset of fever, the patient develops a rash that typically appears first on the face and then spreads to other parts of the body, including the hands and feet (Brown and Leggat, 2016). The cutaneous lesions often present initially as macules and then evolve into papules, vesicles, pustules, crusts, and scabs. MPXV also causes lymphadenopathy (e.g., in the cervical or inguinal region) (Alakunle et al., 2020). For most people, mpox is a self-limited disease, typically lasting two to four weeks and resulting in complete recovery (Thornhill et al., 2022). On the other hand, the study of Immune markers has provided evidence of asymptomatic MPXV infections in individuals vaccinated against smallpox and others who had not been vaccinated (Otter et al., 2023).

Laboratory confirmation of mpox relies primarily on molecular methods, with real-time polymerase chain reaction (qPCR) considered the gold standard for its high sensitivity and specificity in detecting viral DNA in lesion-derived samples, including swabs, crusts, and exudates. Viral DNA can also be detected in other clinical specimens, including saliva and semen, although skin lesion material remains the recommended specimen for laboratory confirmation (Laboratory testing for the monkeypox virus: interim guidance, 23 May 2022, 2026; World Health Organization, 2026). Multiple PCR assays targeting conserved *Orthopoxvirus* genus and MPXV-specific genomic regions, including G2R, F3L, and B6R genes, have been developed and validated for clinical diagnosis and surveillance. In addition to molecular techniques, serological assays and viral culture have historically been used, but their diagnostic value is limited by cross-reactivity among orthopoxviruses and by biosafety requirements, reinforcing PCR as the preferred diagnostic approach (Silva et al., 2023).

Since the onset of the global mpox outbreak in 2022, the disease has been reported in more than 140 countries, with a cumulative total of 168,736 confirmed infections (Pan American Health Organization, 2025). During this global mpox outbreak, numerous commercial real-time PCR protocols were introduced to facilitate rapid laboratory diagnosis. Some of these was the VIASURE MPXV Real Time PCR (VIASURE kit) (Monkeypox virus (MPV) detection kit (PCR fluorescence probing), 2026), the Bioperfectus MPXV Real Time PCR Kit (Bioperfectus kit) (Li et al., 2010, Tan et al., 2023), and the Detection kit for MPXV (PCR-Fluorescence Probing) by Singuway (Singuway kit) (Monkeypox virus (MPV) detection kit (PCR fluorescence probing), 2026). Despite the growing availability of these commercial assays, only a limited number of them have undergone independent clinical validation, and data on the diagnostic performance of the three kits included in this study not only remain scarce but also have never been compared with reference comparator methods like the WHO one in the scientific literature, to the best of our knowledge.

Therefore, the aim of the current study was to conduct a retrospective, comparative diagnostic evaluation of three commercial qPCR kits for the diagnosis of MPXV on an artificially enriched sample set that included 50% MPXV-positive samples.

## Materials and methods

### Study design

This comparative retrospective study was conducted using remnant clinical specimens (n = 300), including 229 skin lesions, 36 vesicular lesions, and 35 serum samples, all collected in Universal Transport Medium (UTM-RT, COPAN, Diagnostics Italy), from patients with suspected MPXV infection. Of the total specimens, 150 were previously confirmed as MPXV DNA positive and 150 were confirmed as MPXV DNA negative or corresponded to other pathogens associated with similar clinical presentations, including Epstein–Barr virus (EBV), Varicella-zoster virus (VZV), and Herpes simplex virus types 1 and 2 (HSV-1 and HSV-2). This sample set, which includes 50% MPXV-positive samples, is biased and does not reflect the real-world prevalence of this pathogen. In this sense, this evaluation is limited to sensitivity, specificity, and agreement between the commercial kits being compared and the reference comparator protocol.

Samples were originally collected between May and December 2022 from male and female patients across all age groups. Skin lesion swabs were collected in 3 mL of UTM-RT, in accordance with standard clinical procedures.

The performance of the evaluated assays was assessed qualitatively by comparison with the WHO MPXV real-time PCR protocol (WHO protocol) as a reference comparator, which was routinely used for diagnostic confirmation (**Table 1**). Specimen integrity was preserved through compliance with internationally accepted recommendations for MPXV sample handling and storage. Following routine diagnostic testing, residual samples were maintained at −20 °C and were not subjected to repeated freeze–thaw cycles prior to analysis, thereby minimizing the risk of nucleic acid degradation.

**Table 1:** Technical specifications of qPCR commercial kits for MPXV detection evaluated in this study.

qPCR protocol/kit	Fluorescence channel	Internal control (fluorescence channel)	Target gene region	Sample volume/total volume (µL)	Limit of detection (LoD)
WHO protocol (reference comparator)	FAM	RNase P (VIC)	G2R_G (generic detection)	5/20	≤5 copies/rxn
Bioperfectus kit	FAM	RNase P (VIC)	F3L	5/25	5 copies/rxn
Viasure kit	FAM	HBB* (HEX/VIC)	G2R_G and F3L	5/20	2 copies/rxn (serum) 8 copies/rxn (throat swabs)
Singuway kit	FAM	IC (HEX/VIC)	Conserved region of MPXV	10/30	500 copies/mL

Summary of the technical specifications of the WHO protocol and the commercial kits evaluated, including target genes, fluorescence channels, reaction conditions, and reported analytical sensitivity (* Hemoglobin Subunit Beta).

This was a blinded study in the sense that neither clinical information nor reference comparator results were available to the performers/readers of the commercial qPCR tests; clinical information and commercial qPCR results were not available to the laboratory staff running the reference comparator qPCR test.

### DNA extraction

DNA was extracted from clinical specimens using the QIAamp DNA Mini Kit (QIAGEN, Hilden, Germany), according to the manufacturer’s instructions. Briefly, 200 µL of each clinical sample was processed, and nucleic acids were eluted in a final volume of 50 µL with elution buffer.

### WHO MPXV real-time PCR protocol (WHO protocol)

Detection of MPXV was performed in accordance with WHO recommendations, which establish nucleic acid amplification testing (NAAT), particularly real-time polymerase chain reaction (qPCR), as the reference method for laboratory confirmation of MPXV infection by detecting virus-specific DNA sequences. Several qPCR protocols have been developed for the detection of orthopoxviruses and MPXV; among these, the assay described by *Li* et al. *(2010)* (Li et al., 2010) is one of the most widely validated and was used as the reference method in this comparative study (**Table 1**).

This protocol comprises three independent qPCR reactions targeting distinct genomic regions: the G2R_G target, which enables generic detection of all MPXV strains; the G2R_WA target, specific to the West African clade (Clade II); and the C3L target, specific to the Congo Basin clade (Clade I). These assays use hydrolysis (TaqMan) probes to ensure high analytical sensitivity and specificity, enabling detection and clade differentiation of MPXV. However, in the present study, the comparative evaluation against the commercial assays was performed exclusively using the generic G2R_G assay, whereas the clade-specific assays were not included in the performance comparison (**Table 1**). However, as part of the routine samples’ workflow, the G2R_WA target assay, (specific to the West African clade or Clade II) and the C3L target assay (specific to the Congo Basin clade or Clade I) were also run. All the MPXV-positive samples included in this study belonged to Clade II, the only one circulating in Ecuador at the time of this study. The PCR thermal cycling for the WHO protocol and all commercial kits was performed on the ABI 7500 Fast system (NWLP and Synnovis) (Li et al., 2010; Laboratory testing for the monkeypox virus: interim guidance, 23 May 2022, 2026).

### Bioperfectus MPXV real time PCR kit (Bioperfectus kit)

This is a CE-IVD (Conformité Européenne – *In Vitro* Diagnostic) marked molecular assay designed to detect MPXV DNA in human clinical samples. It is based on real-time PCR technology. Specific primers and probes are designed based on the F3L gene areas of the MPXV (Yu et al., 2025). In addition, the kit includes amplification of the human housekeeping gene RNase P as an internal control (IC) to monitor specimen adequacy and the efficiency of nucleic acid extraction. The assay has been validated for use with human tonsillar swabs, nasopharyngeal swabs, serum, whole blood, lesion exudate, and scab specimens. The cycle threshold (Ct) value is automatically calculated by instrument software, and samples are considered MPXV-positive when Ct< 40. According to the manufacturer’s instructions for use, the clinical performance of this assay demonstrates a positive percent agreement (PPA) of 97.43% and a negative percent agreement (NPA) of 100% when compared with a Clinical Laboratory Improvement Amendments (CLIA) methodology (XYZ Laboratory, 2009 Ranch Rd. 620 N, STE 325, Austin, TX, USA; CAP-accredited and CLIA-certified laboratory) (Monkeypox virus real time PCR kit, 2026) (**Table 1**).

### VIASURE MPXV real time PCR (VIASURE kit)

This detection kit contains, in each well, all the components necessary for a real-time PCR assay (specific primers/probes, dNTPs, buffer, polymerase) in a stabilized format, as well as an endogenous internal control to monitor the extraction process and/or to assess polymerase activity. The assay targets the G2R_G and F3L genes of MPXV and uses the human housekeeping gene hemoglobin subunit beta (HBB) as an endogenous internal control (EIC). MPXV DNA targets are amplified and detected in the green channel, while the endogenous internal control (IC) is detected in the yellow channel. A positive and a negative control were included in each execution. Following the instructions of use (IFU), these two controls validate the reaction by checking the absence of signal in the negative control well and the presence of signal for MPXV DNA in the positive control well. The EIC verified that the correct result interpretation was performed in accordance with the IFU. Briefly, a sample was considered positive if the Ct value obtained was less than 40 and the internal control showed an amplification signal. A sample was considered negative if it showed no amplification signal in the detection system, while the internal control was positive (Monkeypox virus. Certest biotec - raw materials Diagnostics, 2026) (**Table 1**).

### Detection kit for MPXV (PCR-fluorescence probing) by Singuway (Singuway kit)

This assay is based on real-time fluorescence PCR technology, using specific primers and hydrolysis probes targeting conserved regions of the MPXV genome to qualitatively detect viral DNA. The reaction is performed in a 30 μL final volume containing 10 μL of extracted DNA, with fluorescence acquisition in the FAM channel for MPXV detection and in the HEX/VIC channels for internal control. Thermal cycling is carried out according to the manufacturer’s instructions on compatible real-time PCR platforms. A Ct cut-off value of 38 is established: samples are considered positive when a typical amplification curve is observed in the FAM channel with Ct ≤ 38; Ct values between 38 and< 40 are regarded as indeterminate and require repeat testing, whereas Ct ≥ 40 is interpreted as negative, provided that the internal control amplifies appropriately (Ct ≤ 38) (Monkeypox virus (MPV) detection kit (PCR fluorescence probing), 2026) (**Table 1**).

### Statistical analysis

Statistical analyses were performed using MedCalc^®^ software. The WHO protocol was used as the reference comparator method, and a confirmed MPXV case was defined as a positive result obtained with this reference method. Diagnostic accuracy was assessed using 2×2 contingency tables, from which true-positive (TP), true-negative (TN), false-positive (FP), and false-negative (FN) results were determined. Clinical sensitivity (SE) was calculated as TP/(TP + FN), clinical specificity (SP) as TN/(TN + FP), Cohen’s kappa coefficient (κ) as $\kappa =\left(P_{o}-P_{e})/(1-P_{e}\right)$, where *P_o_* represents the observed agreement and *P_e_* the agreement expected by chance, and overall agreement (ORA) as (TP + TN)/N, where N represents the total number of samples analyzed. All estimates were reported with 95% confidence intervals (95% CI), calculated using standard binomial methods implemented in MedCalc^®^ software.

### Adherence to STARD 2015 reporting guidance

In **Supplementary Table 1**, a checklist for adherence to the STARD 2015 guideline for reporting diagnostic accuracy studies is provided (Bossuyt et al., 2015).

## Results

A total of 300 clinical specimens were analyzed in parallel with the three commercial real-time PCR assays for MPXV detection (**Table 1**). Results of these molecular assays were compared with those obtained in the initial diagnosis conducted in accordance with WHO recommendations. Clade assignment was established using the WHO protocol, including the corresponding clade-specific assay, and all MPXV-positive specimens included in this study were classified as Clade II. Therefore, the diagnostic performance reported herein reflects the evaluation of the commercial assays exclusively in the context of Clade II infections circulating in Ecuador during the study period. Moreover, some specimens were negative for mpox but positive for EBV (n=1) and VZV (n=14).

### Bioperfectus kit vs. WHO protocol

According to the WHO protocol, 150 samples were confirmed as MPXV DNA-positive and 150 as negative, including specimens with other clinically similar infections, such as EBV, VZV, and HSV-1 and HSV-2.

**Figure 1:**
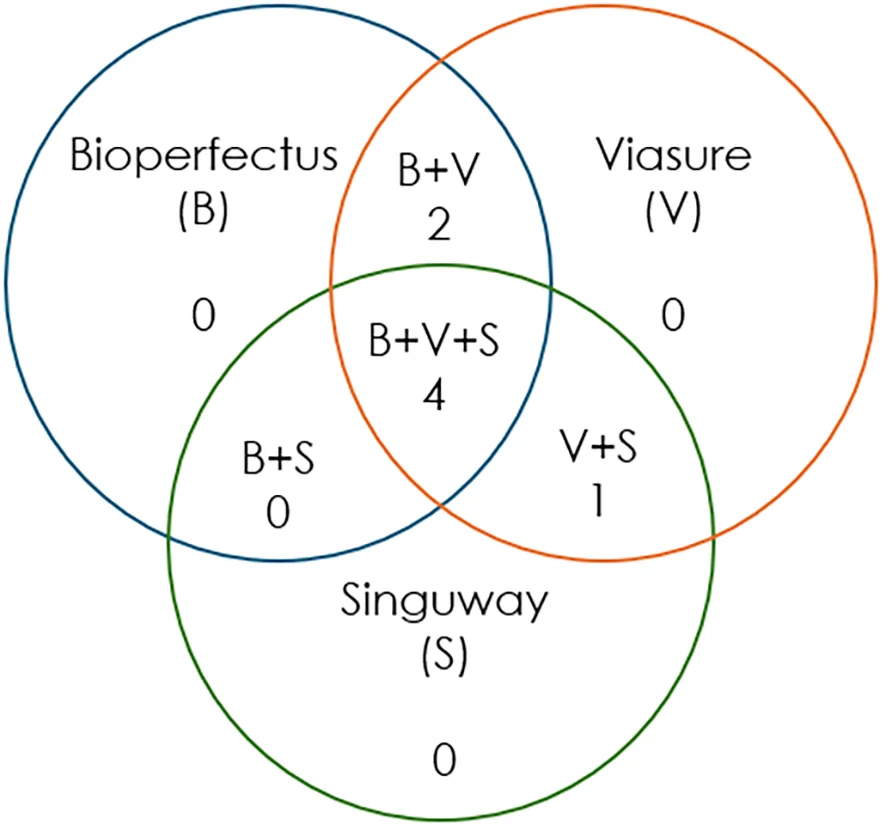
Distribution and overlap of false-negative results among three commercial real-time PCR kits for MPXV detection. Overlap of false-negative MPXV results among the Bioperfectus kit, VIASURE kit and Singuway kit compared with the WHO protocol used as reference comparator. Four samples were missing by all three commercial kits, while three additional samples were shared between two commercial kits. Any of the commercial kits produced unique false-negative results.

The Bioperfectus kit correctly detected 294 of the 300 specimens, with 6 false negatives (WHO Ct values: 36.23; 36.74; 37.12; 37.43; 37.88 and 38.75) and no false positives. Four of these samples share the false-negative status with the other kits evaluated. The distribution of positive and negative results is presented in **Figure 1** and **Table 2**, and **Table 3** summarizes the corresponding diagnostic performance parameters (sensitivity, specificity and Cohen’s kappa coefficient), indicating a high level of accuracy for mpox detection. All false-negative samples were run in triplicate using the Bioperfectus kit and retested using the WHO protocol to rule out sample degradation.

**Table 2:** Confusion matrices of commercial kits versus the WHO protocol (reference comparator protocol) for MPXV detection.

Commercial kit		WHO protocol		
		(+)	(-)	Total
Bioperfectus Kit	(+)	144	0	144
	(-)	6	150	156
	Total	150	150	300
VIASURE Kit	(+)	143	0	143
	(-)	7	150	157
	Total	150	150	300
Singuway kit	(+)	145	0	145
	(-)	5	150	155
	Total	150	150	300

Distribution of MPXV positive (+) and negative (−) results obtained with each commercial kit compared with the WHO protocol (reference comparator) in 300 clinical specimens.

**Table 3:** Diagnostic performance parameters of Bioperfectus kit, VIASURE kit and Singuway kit compared with the WHO protocol (reference comparator).

	Diagnostic performance parameters							
Commercial kit	Overall agreement	TP	TN	FP	FN	SE	SP	κ
Bioperfectus kit	0.98 (0.96 - 0.99)	144	150	0	6	0.96 (0.93 - 0.99)	1.00 (0.98 - 1.00)	0.96 (0.92 - 0.99)
VIASURE kit	0.98 (0.95 – 0.99)	143	150	0	7	0.95 (0.92 – 0.99)	1.00 (0.98–1.00)	0.95 (0.92 - 0.99)
Singuway kit	0.98 (0.97 – 1.00)	145	150	0	5	0.97 (0.94 – 1.00)	1.00 (0.98 – 1.00)	0.97 (0.94 - 1.00)

n=300. True positive (TP), true negative (TN), false positive (FP), false negative (FN), sensitivity (SE), specificity (SP), and Cohen’s Kappa coefficient (κ). The 95% confidence intervals are shown in parentheses.

### VIASURE kit vs. WHO protocol

Following initial classification by the WHO protocol (150 MPXV-positive and 150 MPXV-negative samples), the VIASURE kit demonstrated great diagnostic performance, correctly identifying 293 of 300 specimens. Seven false-negative results were observed (reference assay Ct values ranging from 36.23 to 38.75), with no false-positive findings relative to the reference comparator method (**Figure 1**; **Table 2**). The corresponding diagnostic accuracy parameters (sensitivity, specificity and Cohen’s kappa coefficient), presented in **Table 3**, further support the assay’s analytical robustness for MPXV detection. All false-negative samples were run in triplicate with the Viasure kit and retested with the WHO protocol to exclude sample degradation.

### Singuway kit vs. WHO protocol

Similarly, after the initial characterization (per the WHO protocol), the Singuway kit yielded concordant results in 295 of 300 samples. The five discordant samples corresponded exclusively to false-negative results, with Ct values of 36.23, 36.74, 36.86, 37.88, and 38.75 according to the WHO protocol, while no false-positive results were observed (**Figure 1**; **Table 2**). Notably, four of these false-negative samples were also undetected by the other two commercial assays evaluated, suggesting they contained viral DNA concentrations near the analytical detection limits of those assays. The detailed diagnostic performance parameters (sensitivity, specificity and Cohen’s kappa coefficient), derived from these results, are summarized in **Table 3**, confirming the high agreement of this assay. All false-negative samples were run in triplicate using the Singuway kit and retested with the WHO protocol to exclude sample degradation.

**Figure 2:**
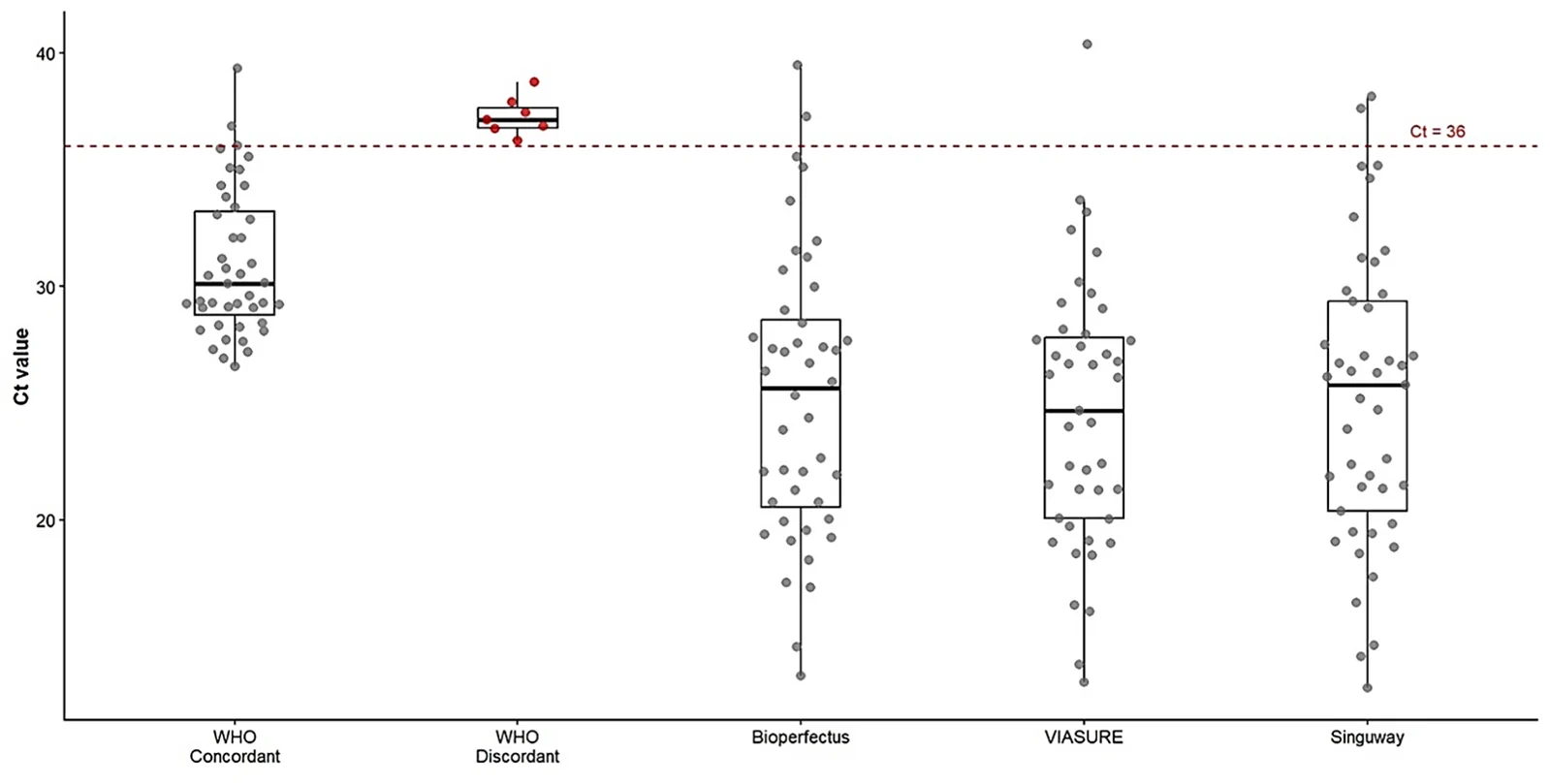
Distribution of cycle threshold (Ct) values among MPXV-positive specimens evaluated with the WHO protocol (reference comparator) and three commercial kits. WHO protocol-positive specimens are shown as concordant-positive (grey), detected by all three commercial kits, and discordant-positive (red), yielding a false-negative result in at least one commercial kit. For Bioperfectus kit, VIASURE kit and Singuway kit only Ct values from specimens with detectable amplification are shown. Boxplots represent the median and interquartile range, and individual points represent individual specimens. The dashed horizontal line indicates Ct = 36.

In **Figure 2**, the distribution of Ct values of the MPXV-positive samples for the WHO protocol and the three evaluated commercial kits is shown. The WHO protocol’s positive samples have been divided into concordant-positive specimens (gray) and those that yielded false-negative results in at least one commercial kits (red). False negative results for the 3 commercial kits were predominantly associated with high Ct values (**Figure 2**).

## Discussion

Since the onset of the global mpox outbreak in 2022, cases have been reported in more than 140 countries, with a cumulative total of 168,736 confirmed infections. The Region of the Americas has been the most affected, accounting for 42.4% of all reported infections (Pan American Health Organization, 2025). Ecuador accumulated 267 confirmed cases between 2022 and 2024. In 2025, cases were reported again, although the regional report confirms their presence without specifying the updated cumulative numbers for each country (Bossuyt et al., 2015; Pan American Health Organization, 2025; Tamami et al., 2025). In this context, the availability and evaluation of commercial real-time PCR assays became essential to support accurate diagnostic in Ecuador, where IVD approvals do not rely on reliable regional agencies, which often lack the infrastructure and staff to conduct evaluations.

In the present study, the three commercial kits assessed demonstrated high sensitivity and specificity and strong agreement with the WHO protocol used as reference comparator. Nevertheless, further experiments with real-world prevalence sample sets are needed to confirm their suitability for routine laboratory detection of MPXV. The sensitivity and specificity observed for the VIASURE kit, Bioperfectus kit and Singuway kits were highly consistent with previously published evaluations. A multicenter study conducted in the United Kingdom by Tan et al. (2023) (Monkeypox virus (MPV) detection kit (PCR fluorescence probing), 2026) reported sensitivity values ranging from 90.0% to 100% and specificity values ranging from 88.2% to 100% for the VIASURE kit, with overall accuracies of 93.2–96.3% across three teaching hospitals. Similarly, the present study demonstrated excellent sensitivity and specificity of the Bioperfectus kit, achieving values of 96.0% and 100% relative to the WHO protocol. These findings are consistent with an independent evaluation conducted by the Robert Koch Institute, which reported high analytical sensitivity (≤5 genome equivalents per reaction) and excellent specificity, with no cross-reactivity against clinically relevant pathogens, including VZV (Michel et al., 2022). Likewise, a recent systematic evaluation of PCR-based diagnostic methods reported pooled sensitivity, and specificity estimates of approximately 99% and 100%, respectively, for the Singuway kit (Unnikrishnan et al., 2026). Consistent with previous reports, the Singuway kit demonstrated excellent sensitivity of 96.6% and specificity of 100% relative to the WHO protocol. Collectively, these findings demonstrate that all three commercial assays show strong agreement in an enriched sample set comprising 50% MPXV-positive samples compared with the WHO protocol as a reference comparator, despite differences in assay design and target regions (**Table 1**). Notably, the few discordant results observed across platforms were exclusively false negatives and were predominantly associated with samples exhibiting high Ct values in the reference comparator assay, as it is clearly shown in **Figure 2**. This observation underscores the importance of appropriate specimen collection and highlights that the sensitivity of commercial qPCR kits may be compromised in samples with very low viral loads. Overall, the concordance between our findings and independent international evaluations would support implementing these assays for routine MPXV diagnosis and surveillance, although further evaluations with real-world MPXV prevalence samples are needed.

An additional finding of this study was the absence of meaningful differences in assay performance between cutaneous and vesicular lesion specimens, as reflected by comparable Ct values across all evaluated platforms. This observation supports current WHO and PAHO recommendations, which identify lesion-derived material as the specimen of choice for MPXV molecular diagnosis due to its consistently high viral DNA burden. Although serum samples were included in the study, their limited number and the generally lower viral loads expected in blood during the symptomatic phase precluded a robust comparative analysis. Furthermore, Ct values generated by the commercial assays showed trends comparable to those obtained with the WHO protocol, indicating a consistent ability to detect specimens spanning a broad range of viral loads. However, absolute Ct values should not be interpreted interchangeably across platforms, as differences in genomic targets, primer–probe composition, amplification chemistry, and analytical algorithms can influence Ct determination. Consequently, Ct values are most appropriately interpreted within the context of each individual assay rather than as directly comparable quantitative measures between diagnostic platforms.

Our study has limitations that we would like to acknowledge. First, this study was carried out with an artificially enriched sample set that included 50% MPXV-positive samples, so only information related to sensitivity, specificity, and agreement between methods could be derived from these results; further evaluations, including sample sets with true MPXV prevalence, are needed for a complete clinical performance evaluation. Second, by the time this study was carried out, no cases of Clade I MPXV had been detected in Ecuador. In fact, the first case of Clade I was as recent as April 2026; so, this evaluation study was carried out exclusively with Clade II MPXV samples, and we cannot entirely rule out that the diagnostic performance of the three commercial kits included in this study may differ for Clade I MPXV. From a public health perspective, the introduction of this emerging lineage highlights the importance of continuously evaluating the performance of molecular diagnostic tools under local epidemiological conditions. The three commercial kits evaluated in this study were selected because they were among the first CE-IVD molecular platforms available to the National Reference Laboratory during the 2022 mpox outbreak and represented suitable candidates for integration into the national diagnostic network. So far, this kind of study provides valuable information about the sensitivity and specificity of commercial kits to inform the selection of molecular assays in clinical and public health laboratories.

In conclusion, the three evaluated commercial kits had a high sensitivity and specificity compared to the WHO protocol for MPXV diagnosis. In the context of the 2022–2023 global mpox outbreak and the subsequent implementation of molecular diagnostic capacity in low- and middle-income countries such as Ecuador, the availability of commercial kits that strongly agrees with in-house protocols, such as the WHO protocol, supports more accurate diagnosis of MPXV and surveillance of mpox cases.

## Data Availability

The original contributions presented in the study are included in the article/**Supplementary Material**. Further inquiries can be directed to the corresponding author.

## Supplementary material

Supplementary Table 1 Checklist mapping each of the 30 essential items of the STARD 2015 statement to its current location in the manuscript. “Reported” indicates the item is adequately addressed; “N/A” indicates the item does not apply to this study design.
