# Supplementary material for: A retrospective comparative diagnostic evaluation of three commercial qPCR kits for the diagnosis of mpox virus on an enriched sample set
Source: Front Cell Infect Microbiol. 2026 Sep 11;16:1831182. doi: 10.3389/fcimb.2026.1831182 (PMC13612254; doi:10.3389/fcimb.2026.1831182)
Supplement: Supplementary file 1 [file Table1.docx]

**Supplementary table 1. Checklist mapping each of the 30 essential items of the STARD 2015 statement to its current location in the manuscript. "Reported" indicates the item is adequately addressed; "N/A" indicates the item does not apply to this study design.**

| **No.** | **Section and topic** | **STARD 2015 item** | **Status** | **Location in manuscript** | **Remarks** |
| --- | --- | --- | --- | --- | --- |
| 1 | Title or abstract | Identification as a study of diagnostic accuracy using at least one measure of accuracy (e.g. sensitivity, specificity, predictive values, or AUC) | **Reported** | Title | Title clearly identifies the study as a comparative diagnostic evaluation. |
| 2 | Abstract | Structured summary of study design, methods, results, and conclusions (see STARD for Abstracts) | **Reported** | Abstract | All IMRAD elements are present, but they are written as a single paragraph. |
| 3 | Introduction | Scientific and clinical background, including the intended use and clinical role of the index test | **Reported** | Introduction, paragraphs 1–4 | Background on MPXV biology, transmission and diagnostic role of qPCR is provided. |
| 4 | Introduction | Study objectives and hypotheses | **Reported** | Introduction, final paragraph | Objective is stated; no explicit a priori hypothesis. |
| 5 | Study design | Whether data collection was planned before the index test and reference standard were performed (prospective) or after (retrospective) | **Reported** | Methods: Study design | "This comparative retrospective study was conducted using remnant clinical specimens…" |
| 6 | Participants | Eligibility criteria | **Reported** | Methods: Study design | Samples from patients previously tested positive for MPXV |
| 7 | Participants | On what basis potentially eligible participants were identified (symptoms, results from previous tests, registry, etc.) | **Reported** | Methods: Study design | Based on prior WHO qPCR classification (150 positive / 150 negative). |
| 8 | Participants | Where and when potentially eligible participants were identified (setting, location, dates) | **Reported** | Methods: Study design | Collection dates given (May–December 2022). The testing site/setting (National Reference Laboratory). |
| 9 | Participants | Whether participants formed a consecutive, random, or convenience series | **Reported** | Methods: Study design | Convenience sample set enriched in samples from patients previously tested MPXV positive |
| 10a | Test methods | Index test, in sufficient detail to allow replication | **Reported** | Methods, kit-specific subsections (Bioperfectus / VIASURE / Singuway) | Target genes, channels, controls and cut-offs described for each kit. |
| 10b | Test methods | Reference standard, in sufficient detail to allow replication | **Reported** | Methods: WHO reference molecular assay | G2R_G/G2R_WA/C3L targets, Li et al. 2010 protocol, platform (ABI 7500 Fast) described. |
| 11 | Test methods | Rationale for choosing the reference standard (if alternatives exist) | **Reported** | Methods: WHO reference molecular assay | Cited as "one of the most widely validated protocols”. |
| 12a | Test methods | Definition of and rationale for test positivity cut-offs of the index test, distinguishing pre-specified from exploratory | **Reported** | Methods, kit-specific subsections | Ct < 40 (Bioperfectus, VIASURE) and Ct ≤ 38 (Singuway), all manufacturer-specified (pre-specified). |
| 12b | Test methods | Definition of and rationale for test positivity cut-offs of the reference standard, distinguishing pre-specified from exploratory | **Reported** | Methods: WHO reference molecular assay | G2R_G/G2R_WA/C3L targets, Li et al. 2010 protocol, platform (ABI 7500 Fast) described. |
| 13a | Test methods | Whether clinical information and reference standard results were available to the performers/readers of the index test | **Reported** | Methods: Study design |  |
| 13b | Test methods | Whether clinical information and index test results were available to the assessors of the reference standard | **Reported** | Methods: Study design |  |
| 14 | Analysis | Methods for estimating or comparing measures of diagnostic accuracy | **Reported** | Methods: Statistical analysis | Sensitivity, specificity, Cohen's kappa, overall agreement and 95% CI methods described (MedCalc®). |
| 15 | Analysis | How indeterminate index test or reference standard results were handled | **Reported** | Methods, kit-specific subsections and WHO reference molecular assay | Indeterminate handling (Ct 38–<40, retested) is only specifically described only for Singuway kit; for other kits and WHO, only positive and negative results were reported for every sample. |
| 16 | Analysis | How missing data on the index test and reference standard were handled | **N/A** | — | Not applicable. |
| 17 | Analysis | Any analyses of variability in diagnostic accuracy, distinguishing pre-specified from exploratory | **Reported** | Discussion: (specimen-type comparison) | The cutaneous-vs-vesicular Ct comparison appears in the Discussion. |
| 18 | Analysis | Intended sample size and how it was determined | **Reported** | — | n=300 (150 positive / 150 negative). |
| 19 | Participants (Results) | Flow of participants, using a diagram | **N/A** | — | Not applicable. |
| 20 | Participants (Results) | Baseline demographic and clinical characteristics of participants | **Reported** | Results | It is mentioned: male and female patients across all age groups. |
| 21a | Participants (Results) | Distribution of severity of disease in those with the target condition | **N/A** | — | Not applicable. |
| 21b | Participants (Results) | Distribution of alternative diagnoses in those without the target condition | **Reported** | Results | EBV (n=1) and VZV (n=14) explicitly reported; HSV-1/2 mentioned in methods as part of the negative panel. |
| 22 | Participants (Results) | Time interval and any clinical interventions between index test and reference standard | **Reported** | Results | Results analyzed in parallel. |
| 23 | Test results | Cross tabulation of the index test results (or their distribution) by the results of the reference standard | **Reported** | Table 2 | 2×2 confusion matrices provided for all three kits vs WHO protocol. |
| 24 | Test results | Estimates of diagnostic accuracy and their precision (e.g. 95% CI) | **Reported** | Table 3 | Sensitivity, specificity, kappa and overall agreement with 95% CI for all three kits. |
| 25 | Test results | Any adverse events from performing the index test or the reference standard | **N/A** | — | Not applicable. Retrospective testing of archived residual specimens; no patient-facing procedure. |
| 26 | Discussion | Study limitations, including sources of potential bias, statistical uncertainty, and generalisability | **Reported** | Discussion, limitations paragraph | Enriched sample set, Clade II-only samples discussed. |
| 27 | Discussion | Implications for practice, including the intended use and clinical role of the index test | **Reported** | Discussion / Conclusion | Implications for national diagnostic networks in Ecuador/LMIC settings discussed. |
| 28 | Other information | Registration number and name of registry | **N/A** | — | Not applicable. |
| 29 | Other information | Where the full study protocol can be accessed | **N/A** | — | Not applicable. |
| 30 | Other information | Sources of funding and other support; role of funders | **N/A** | — | Not applicable. |
